# Synthesis and Antifungal Activity of 5-Chloro-6-Phenyl-pyridazin-3(2*H*)-one Derivatives 

**DOI:** 10.3390/molecules14093676

**Published:** 2009-09-18

**Authors:** Jian Wu, Baoan Song, Hongjun Chen, Pinaki Bhadury, Deyu Hu

**Affiliations:** Key Laboratory of Green Pesticide and Agriculture Bioengineering, Ministry of Education, Research and Development Center for Fine Chemicals, Guizhou University, Guiyang 550025, China; E-Mails: jianwu2691@yahoo.com.cn (J.W.); chj020726@163.com (H-J.C.); bhadury@gzu.edu.cn (P.B.); dyhu@gzu.edu.cn (D.H.)

**Keywords:** pyridazine derivatives, synthesis, antifungal activity

## Abstract

An effective method has been developed for the preparation under mild conditions of novel pyridazine derivatives from the easily accessible starting materials mucochloric acid and benzene. All the synthesized compounds were fully characterized and some of them displayed good antifungal activities against *G. zeae*, *F. oxysporum* and *C. mandshurica* in preliminary antifungal activity tests.

## 1. Introduction

**Scheme 1:**
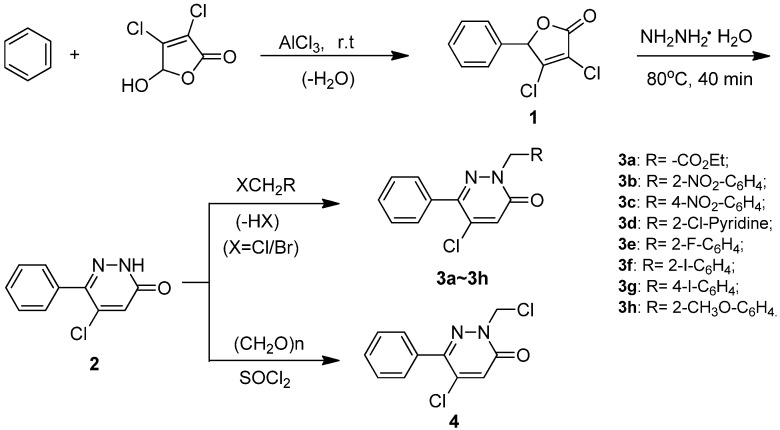
Synthetic route to **3a-3h** and **4**.

**Scheme 2:**
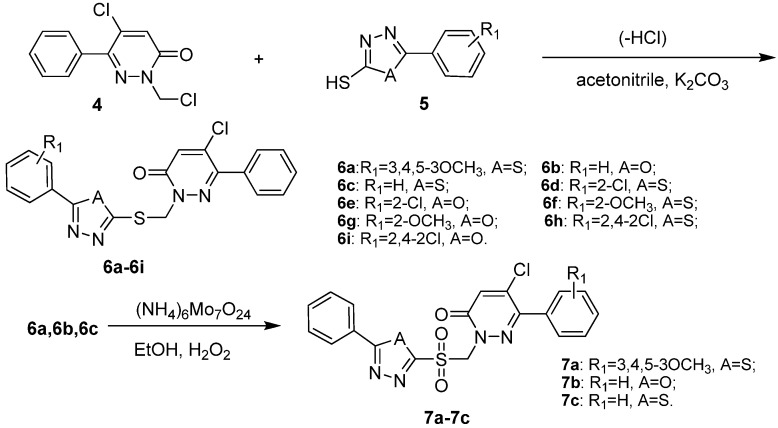
Synthetic route to **6a-6i** and **7a-7c**.

Many pyridazine derivatives are well known to possess a wide range of bioactivities and are often employed as plant virucides [1,2], antitumor agents [3,4,5], fungicides [6,7,8], insecticides [9,10], herbicides [11,12,13,14,15] and anti-inflammatory agents [16,17]. They have immense potential in agricultural science as plant growth regulators and crop protection agents. Several derivatives of these compounds incorporating 1,3,4-thiadiazole, 1,3,4-oxadiazole and oxazolidin-2-one rings have been shown to display moderate to good antifungal activities [18,19,20]. Introduction of methyl *N*-methoxy-*N*-[2-(1,6-dihydro-1-substitued-6-oxo-pyridazin-3-yloxymethyl) phenyl] carbonate base into pyrazole derived pyraclostrolin has resulted in the development of compounds with appreciable fungicidal activities against *P. oryzae*, *B. cinerea* and *E. graminis* [21]*.* In addition, a series of halogen and aryl substituted pyridazine derivatives synthesized by Akio and Trah [22,23] exhibited high fungicidal activities at a concentration of 50 µg/mL. Since oxadiazole and thiadiazole derivatives are associated with good antifungal characteristics [24,25,26], we envisioned that modification of 5-chloro-6-phenylpyridazin-3(2*H*)-one by suitable substituent replacement at the *N*-2 position, followed by incorporation of 1,3,4-thiadiazole and 1,3,4-oxadiazole scaffolds into the pyridazin-3(2*H*)-one ring through a “-CH_2_S-” moiety could result into the formation of lead structures with potent activity. The pyridazines **3a-3h** and **4** without the presence of any oxadiazole and thiadiazole moieties were prepared from mucochloric acid and benzene as depicted in Scheme 1. Then 12 novel pyridazines **6a-6i** and **7a-7c** derived from **4** incorporating the above structural features were prepared. The synthetic sequence is shown in Scheme 2. All 21 compounds synthesized were unequivocally characterized by IR, NMR and elemental analysis. Preliminary antifungal activity tests showed that most of the compounds exhibited inhibitory activity against *G. zeae*, *F. oxysporum* and *C. mandshurica* to a certain extent. Among them, compounds **3e**, **3h**, **7b**, **7c** exhibited slightly superior or similar activities as compared to the commercial agent hymexazol on the corresponding fungi.

## 2. Results and Discussion

### 2.1. Chemistry

3,4-Dichloro-5-phenylfuran-2(5*H*)-one (**1**) was synthesized via Friedel-Crafts reaction by employing mucochloric acid and benzene as starting materials in the presence of the Lewis acid AlCl_3_, in accordance with the known synthetic protocols described in the literature [27,28].

Preparation of the compound 5-chloro-6-phenylpyridazin-3(2*H*)-one (**2**) has been described previously [27,29], but unfortunately in our hands under the same reaction conditions the yield was much lower (46%) compared to the one reported in the literature. It was observed that the type of solvent, reaction temperature and reaction time were the most important parameters affecting the purity and yield of the final product. The best result, affording a yield of 68%, was achieved when the reaction was perfomed in the solvent DMF at 80°C for 40 min.

5-Chloro-6-phenyl-2-substituted-pyridazin-3(2H)-ones **3a-3h** were then conveniently prepared in good yields by treatment of 5-chloro-6-phenylpyridazin-3(2*H*)-one (**2**) with halide (XCH_2_R, X = Cl or Br) in acetone, acetonitrile or *N*,*N*-dimethylformamide. Among these solvents, however, acetone provided the best results and the reaction could be successfully conducted at room temperature. The reaction was much faster and high yielding when X was bromine instead of chlorine. This kind of substitution at the nitrogen atom of **2** in the presence of a base was also previously investigated [28] by Estevez *et al*. 

Compound **2** was also separately reacted (Scheme 1) with paraformaldehyde and thionyl chloride in benzene to afford 5-chloro-2-(chloromethyl)-6-phenylpyridazin-3(2*H*)-one (**4**) in a single step in 73% yield. In comparison with the two-step process as reported in the literature [30], the operation was much more convenient and the reaction time was significantly shortened. 

Finally, the pyridazine derivatives **6a**-**6i** with 1,3,4-thiadiazole or 1,3,4-oxadiazole moieties were easily obtained in 60-80% yields by the reaction of 5-chloro-2-(chloromethyl)-6-phenylpyridazin-3(2*H*)-one (**4**) with **5** as depicted in Scheme 2. As the progress of the reaction was monitored by TLC, the possibility of a side reaction through the chlorine atom at C-5 position of pyradazine ring could not be ruled out. The reaction time and temperature were critical for this reaction. In general, the products were obtained under mild conditions at 50 °C with a reaction time of 3-4 hours. The compounds **6a-6c** were eventually oxidized to **7a-7c** by H_2_O_2_ and (NH_4_)_6_Mo_7_O_24_ as shown in Scheme 2.

The structures of the compounds **1**, **2**, **3a-3h**, **4**, **6a-6i** and **7a-7c** were established on the basis of their spectroscopic data. The IR spectra showed absorption bands around 3,049-3,099 cm^-1^ for the Ar-H stretching vibrations and near 1,662-1,678 cm^-1^ for the presence C=O functional groups. In the ^1^H- NMR spectra of the pyradazine derivatives, the 4-*H* signal appeared as a singlet in 7.14-7.26 ppm range, while the Ar-H peaks of all the derivatives were observed near 6.64-8.40 ppm as a multiplet. The CH_2_ peaks were observed as singlets in 5.30-6.31 ppm range.

### 2.2. Antifungal activity bioassay

The *in vitro* antifungal screening data of the pyridazine derivatives are provided in Table 1. It was observed that these synthesized compounds showed weak to good antifungal activities against the tested fungi at 50 μg/mL. Compounds **3d**, **3e** and **6b** were shown to inhibit the growth of *G. zeae* at 45.1%, 43.8%, and 40.4%, respectively; compounds **3d**, **3f** and **7c** exhibited good activities on *F. oxysporum* at 38.2%, 44.2% and 43.1%, respectively while compounds **3d**, **3e** and **3h** inhibited the growth of *C. mandshurica* at 43.5%, 40.6% and 47.8%, respectively. These figures were slightly lower than those of hymexazol. It should be noted that compounds **3h**, **7b** and **7c** showed good activities on *G. zeae* at 50.3%, 57.9% and 60.5%, respectively; compounds **3e** and **3h** exhibited the growth of *F. oxysporum* at 53.2% and 50.9% respectively and compound **7c** exhibited good activity on *C. mandshurica*. Amongst the four compounds **3e**, **3h**, **7b**, **7c** that exhibited similar activities as that of hymexozole on their corresponding fungi, the last two showed considerable promise. Although, a definite structure activity relationship could not be established with the limited experimental data and available compounds, it appears that incorporation of oxadiazole or thiadiazole unit through thiol **5** into parent pyridazine derivative and subsequent oxidation of the resulting product to sulfone **7** might have a positive influence to enhance antifungal activity of the designed compounds.

**Table 1:** Inhibition effects of pyridazine derivatives on phytopathogenic fungi.

Compd.( 50 µg/mL)	*G. zeae*	*F. oxysporum*	*C. mandshurica*
**3a**	7.5	12.3	4.5
**3b**	8.3	18.9	19.2
**3c**	33.7	25.5	26.7
**3d**	45.1	38.2	43.5
**3e**	43.8	53.2	40.6
**3f**	24.2	44.2	33.8
**3g**	22.3	18.7	15.9
**3h**	50.3	50.9	47.8
**4**	17.8	10.7	12.1
**6a**	12.1	9.9	13.2
**6b**	40.4	27.1	30.0
**6c**	16.2	9.4	13.8
**6d**	26.2	3.4	0.8
**6e**	29.0	9.14	1.5
**6f**	16.6	8.1	6.2
**6g**	35.3	32.9	28.3
**6h**	11.2	-3.5	-2.5
**6i**	22.1	3.8	2.0
**7a**	35.5	32.6	34.6
**7b**	57.9	35.3	26.3
**7c**	60.5	43.1	52.1
hymexazol	50.4	52.4	54.1

## 3. Experimental

### 3.1. General

Unless otherwise stated, all the reagents and reactants were purchased from commercial suppliers; melting points were uncorrected and determined on a XT-4 binocular microscope (Beijing Tech Instrument Co., China). The ^1^H-NMR and ^13^C-NMR spectra were recorded on a JEOL ECX 500 NMR spectrometer at room temperature operating at 500 MHz for ^1^H-NMR and 125 MHz for ^13^C-NMR, using CDCl_3_, CD_3_COCD_3_ or DMSO as solvents and TMS as an internal standard; infrared spectra were recorded in KBr on a Bruker VECTOR 22 spectrometer; elemental analysis was performed on an Elemental Vario-III CHN analyzer. The course of the reactions was monitored by TLC; analytical TLC was performed on silica gel GF_254_; column chromatographic purification was carried out using silica gel. 5-Subsititued phenyl-1,3,4-thiadiazoles (or oxadiazole)-2-thiols **5** were prepared according to the literature procedure [25,26] from substituted benzoic acid as the starting material through esterification, hydrazidation, salt formation, and cyclization. All compounds were synthesized under mild conditions with moderate yields.

### 3.2. Preparation of 3, 4-dichloro-5-phenylfuran-2(5H)-one *(**1**)*

Mucochloric acid (33.0 g) was slowly added with stirring to a mixture of benzene (160 mL) and anhydrous aluminum chloride (40.0 g). After completion of the addition, stirring was continued for 3 h at room temperature. After addition of ice (60.6 g) and conc. HC1 (128 mL), the resulting mixture was extracted with benzene (4 × 50 mL). The combined extract was dried on anhydrous Na_2_SO_4_ and concentrated under vacuum to afford a crystalline solid which was filtered off and recrystallized from methanol, m.p. 75-77 °C (lit. [27], m.p. 79-81 °C); yield 60%. 

### 3.3. Preparation of 5-chloro-6-phenylpyridazin-3(2H)-one *(**2**)*

Hydrazine hydrate (80%, 6.0 g) was slowly added to a solution of 3,4-dichloro-5- phenylfuran-2(5*H*)-one (5.0 g) dissolved in *N*,*N*-dimethylformamide (30 g). The resulting solution was stirred at 80 °C for 40 min; after cooling, the mixture was added to water (150 mL) to give a precipitate which was filtered off, washed with water and recrystallized from dioxane to give a yellow solid. Yield, 68%; m.p. 231-232 °C (lit. [27], m.p. 230-231°C). 

### 3.4. Preparation of 5-chloro-6-phenyl-2-substitutedpyridazin-3(2H)-ones ***3a-3h***

To a well stirred solution of 5-chloro-6-phenylpyridazin-3(2*H*)-one (1.00 mmol) in acetone (8 mL) anhydrous potassium carbonate (0.5 g) and halide (1.00 mmol) were added. The mixture was stirred at room temperature and monitored by TLC. After completion of the reaction, the solid was filtered; the solvent was evaporated and the crude product was purified by preparative TLC with a mixture of petroleum ether and ethyl acetate (v:v = 1:1) as developing solvent to give title compounds **3a-3h**.

*Ethyl 2-(4-chloro-6-oxo-3-phenylpyridazin-1(6H)-yl)acetate* (**3a**): Light yellow solid, yield 72%; m.p. 77.0-78.5 °C; ^1^H-NMR (CDCl_3_): *δ* 7.43-7.57 (m, 5H, Ph-H), 7.16 (s, 1H, pyridazine-H), 4.92 (s, 2H, CH_2_), 2.25 (q, *J* = 7.45, 2H, CH_2_), 1.29 (t, *J* = 6.9Hz, 3H, CH_3_); ^13^C-NMR (CDCl_3_): *δ* 167.06, 158.85, 145.82, 140.48, 133.38, 129.73, 129.29, 128.79, 128.35, 62.00, 53.22, 14.21; IR: *ν* 3059.1, 3034.0, 2900.0, 1737.8, 1678.0 cm^-1^; Anal. Calc. for C_14_H_13_ClN_2_O_3_: C 57.44%, H 4.48%, N 9.57%. Found: C 57.73%, H 4.23%, N 9.98%.

*2-(2-Nitrobenzyl)-5-chloro-6-phenylpyridazin-3(2H)-one* (**3b**): White solid, yield 70%, m.p. 116-118 °C; ^1^H-NMR (CDCl_3_): *δ* 7.20-8.10 (m, 9H, Ph-H), 7.18 (s, 1H, pyridazine-H), 5.79 (s, 2H, CH_2_); ^13^C-NMR (CDCl_3_): *δ* 158.92,148.65, 146.03, 140.27, 133.75, 133.22, 131.06, 129.80, 129.34, 129.24, 129.04, 128.85, 128.36, 125.36, 152.55; IR: *ν* 3030.0, 2943.3, 1662.6 cm^-1^; Anal. Calc. for C_17_H_12_ClN_3_O_3_: C 59.75%, H 3.54%, N 12.30%. Found: C 59.43%, H 3.51%, N 12.02%.

*2-(4-Nitrobenzyl)-5-chloro-6-phenylpyridazin-3(2H)-one* (**3c**): White solid, yield 72%, m.p. 143-145°C; ^1^H-NMR (CDCl_3_): *δ* 7.46-8.20 (m, 9H, Ph-H), 7.15 (s, 1H, pyridazine-H), 5.43 (s, 2H, CH_2_); ^13^C-NMR (CDCl_3_): *δ* 158.74, 147.86, 145.99, 142.67, 140.24, 133.33, 129.86, 129.78, 129.24, 129.07, 128.42, 124.02, 56.64; IR: *ν* 3020.0, 2960.1, 1662.6 cm^-1^; Anal. Calc. for C_17_H_12_ClN_3_O_3_: C 59.75%, H 3.54%, N 12.30%. Found: C 59.85%, H 3.86%, N 12.21%.

*5-Chloro-2-[(6-chloropyridin-3-yl)methyl]-6-phenylpyridazin-3(2H)-one* (**3d**): Light yellow solid, yield 68%, m.p. 121-123 °C; ^1^H-NMR (acetone-*d*_6_): *δ* 7.39-8.46 (m, 8H, Ar-H), 7.19 (s, 1H, pyridazine-H), 5.35 (s, 2H, CH_2_); ^13^C-NMR (acetone-*d*_6_): *δ* 158.22, 150.55, 150.11, 145.20, 139.72, 139.47, 134.01, 131.53, 129.45, 129.35, 128.69, 128.20, 124.16, 51.69: IR: *ν* 3076.4, 3059.1, 2950.0, 1654.9 cm^-1^; Anal. Calc. for C_16_H_11_Cl_2_N_3_O: C 57.85%, H 3.34%, N 12.65%. Found: C 57.84%, H 3.63%, N 12.14%.

*2-(2-Fluorobenzyl)-5-chloro-6-phenylpyridazin-3(2H)-one* (**3e**): White crystals, yield 60%, m.p. 91.7-93.8 °C; ^1^H-NMR (CDCl_3_): *δ* 7.05-7.58 (m, 10H, Ar-H) , 5.44 (s, 2H, CH_2_); ^13^C-NMR (CDCl_3_): *δ*162.06, 160.08, 158.89, 139.80, 130.80, 130.00, 129.94, 129.61, 129.29, 128.92, 128.31, 124.30, 122.68, 115.75, 115.58, 49.29; IR: *ν* 3075.0, 3024.3, 1662.6 cm^-1^; Anal. Calc. for C_17_H_12_ClFN_2_O: C 64.87%, H 3.84%, N 8.90%. Found: C 64.84%, H 3.80%, N 8.87%.

*2-(2-Iodobenzyl)-5-chloro-6-phenylpyridazin-3(2H)-one* (**3f**): Light yellow solid, yield 70%, m.p. 78.1-79.6 °C; ^1^H-NMR (acetone-*d*_6_): *δ* 7.03-7.90 (m, 9H, Ph-H), 7.24 (s, 1H, Pyridazine-H), 5.34 (s, 2H, CH_2_); ^13^C-NMR (acetone-*d*_6_): *δ* 158.34, 145.00, 139.61, 139.41, 138.67, 134.02, 129.49, 129.39, 129.35, 128.67, 128.63, 128.53, 128.15, 97.82, 59.15; IR: *ν* 3041.7, 3026.4, 1668.4 cm^-1^; Anal. Calc. for C_17_H_12_ClIN_2_O: C 48.31%, H 2.86%, N 6.63%: Found C 48.63%, H 2.37%, N 6.19%.

*2-(4-Iodobenzyl)-5-chloro-6-phenylpyridazin-3(2H)-one* (**3g**): White solid, yield 79%, m.p. 75.1-76.9 °C; ^1^H-NMR (CDCl_3_): *δ* 7.21-7.67 (m, 9H, Ph-H), 7.03-7.90 (m, 9H, Ph-H),5.28 (s, 2H, CH_2_), 7.24 (s, 1H, pyridazine-H); ^13^C-NMR (CDCl_3_): *δ* 158.78, 145.51, 139.88, 137.88, 135.30, 133.53, 131.03, 129.98, 129.71, 129.29, 128.37, 94.18, 54.91; IR: *ν* 3057.2, 3026.3, 2950.5, 1666.5 cm^-1^; Anal. Calc. for C_17_H_12_ClIN_2_O: C 48.31%, H 2.86%, N 6.63%. Found C 48.14%, H 2.42%, N 6.36%.

*2-(2-Methoxybenzyl)-5-chloro-6-phenylpyridazin-3(2H)-one* (**3h**): White solid, yield 70%, m.p. 92.4-94.0 °C; ^1^H-NMR (acetone-*d*_6_): *δ* 7.18 (s, 1H, pyridazine-H), 6.83-7.57 (m, 9H, Ph-H), 5.33 (s, 2H, CH_2_), 3.83 (s, 3H, OCH_3_); ^13^C-NMR (acetone-*d*_6_): *δ* 158.36, 157.31, 144.47, 139.01, 134.21, 129.29, 129.31, 128.81, 128.45, 128.13, 124.50, 120.35, 110.64, 55.09, 49.75; IR: *ν* 3061.0, 2953.0, 2833.4 1666.5 cm^-1^; Anal. Calc. for C_18_H_15_ClN_2_O_2_: C 66.16%, H 4.63%, N 8.57%. Found: C 66.67%, H 4.64%, N 8.60%.

### 3.5. Preparation of 5-chloro-2-(chloromethyl)-6-phenylpyridazin-3(2H)-one *(**4**)*

To a round bottomed flask containing dry benzene (50 mL) was added 5-chloro-6- phenylpyridazin-3(2*H*)-one (**4,** 2.2 g), paraformaldehyde (1.5 g) and thionyl chloride (3 mL). The resulting mixture was heated under reflux for 1 hour, then cooled to room temperature and filtered. The filtrate was evaporated to dryness under reduced pressure to give a crude solid, which was recrystallised from anhydrous ethanol to afford white crystals, yield 73%, m.p. 108-110 °C; ^1^H-NMR (CDCl_3_): *δ* 7.48-7.59 (m, 6H, Ar-H), 5.86 (s, 2H, CH_2_); ^13^C-NMR (CDCl_3_): *δ* 157.96, 146.92, 141.11, 132.96, 130.06, 129.27, 129.18, 128.45, 57.83; IR: *ν* 3057.1, 3024.3, 2933.7, 1674.21 cm^-1^; Anal. Calc. for C_11_H_8_Cl_2_N_2_O: C 51.79%, H 3.16%, N 10.98%. Found: C 51.82%, H 3.24%, N 11.03%.

### 3.6. Preparation of 5-chloro-6-phenyl-2-[[5-substituted-phenyl-1,3,4-thiadiazol- (or oxadiazol)-2-ylthio]methyl]pyridazin-3(2H)-ones ***6a-6i***

To a suspension of 5-subsititued phenyl-1,3,4-thiadiazole (or oxadiazole)-2-thiol **5** in acetone (or acetonitrile), potassium carbonate (0.5 g) and 5-chloro-2-(chloromethyl)-6-phenylpyridazin-3(2H)-one (**4**, 2 mmol) were added successively; the mixture was stirred and refluxed for 2 h. The solid was filtered off, and the mother liquor was evaporated to give the crude product, which was purified by chromatography on silica using a mixture of petroleum ether and ethyl acetate (v/v = 2:1) as an eluant to provide the target compounds.

*5-Chloro-6-phenyl-2-[[5-(3,4,5-trimethoxyphenyl)-1,3,4-thiadiazol-2-ylthio]methyl]pyridazin-3(2H)-one* (**6a**), White solid, yield 65%, m.p. 97-99 °C; ^1^H-NMR (DMSO-*d*_6_): *δ* 7.25-7.49 (m, 7H, Ph-H), 7.17 (s, 1H, pyridazine-H), 5.85 (s, 2H, CH_2_), 3.79-3.89 (m, 9H, OCH_3_); ^13^C-NMR (DMSO-*d*_6_): *δ* 170.68, 166.57, 161.66, 157.64, 157.60, 154.09, 145.25, 140.07, 129.52, 129.27, 129.21, 128.57, 128.55, 128.13, 128.08, 125.08, 105.18, 104.22, 59.94, 55.91, 55.88, 55.12; IR: *ν* 3057.1, 3024.4, 2935.7, 2833.4, 1675.1 cm^-1^; Anal. Calc. for C_22_H_19_ClN_4_O_4_S_2_: C 52.53%, H 3.81%, N 11.14%. Found: C 52.94%, H 3.29%, N 11.62%.

*5-Chloro-6-phenyl-2-[(5-phenyl-1,3,4-oxadiazol-2-ylthio)methyl]pyridazin-3(2H)-one* (**6b**): Light yellow solid, yield 75% , 70.1-71.5 °C; ^1^H-NMR (acetone-*d*_6_): *δ* 7.32-7.93(m, 10H, Ph-H), 7.22 (s, 1H, pyridazine-H), 5.85 (s, 2H, CH_2_); ^13^C-NMR (acetone-*d*_6_): *δ* 166.54, 161.58, 157.67, 145.53, 133.53, 132.00, 129.57, 129.29, 129.26, 128.59, 128.15, 126.69, 53.37; IR: *ν* 3062.9, 3035.9, 2968.4, 1662.6 cm^-1^; Anal. Calc. for C_19_H_13_ClN_4_O_2_S: C 53.21%, H 3.06%, N 13.06%. Found: C 53.00%, H 3.76%, N 12.64%.

*5-Chloro-6-phenyl-2-[[5-phenyl-1,3,4-thiadiazol-2-ylthio]methyl]pyridazin-3(2H)-one* (**6c**): Light yellow solid, yield 75%, m.p. 87-88 °C; ^1^H-NMR (acetone-*d*_6_): *δ* 7.34-7.91 (m, 10H, Ph-H), 7.23 (s, 1H, pyridazine-H), 5.86 (s, 2H, CH_2_); ^13^C-NMR (acetone-*d*_6_): *δ* 170.69, 162.06, 157.59, 145.24, 140.06, 133.63, 131.51, 129.94, 129.50, 129.46, 129.25, 128.56, 128.12, 127.70, 53.01; IR: *ν* 3082.2, 3020.5, 2947.3, 1670.4 cm^-1^; Anal. Calc. for C_19_H_13_ClN_4_OS_2_: C 55.27%, H 3.17%, N 13.57%. Found: C 55.74%, H 3.63%, N 13.92%.

*5-Chloro-2-[[5-(2-chlorophenyl)-1,3,4-thiadiazol-2-ylthio]methyl]-6-phenylpyridazin-3(2H)-one* (**6d**): Yellow solid, yield 76%, m.p. 116.9-118.4 °C; ^1^H-NMR (DMSO-*d*_6_) *δ*: 7.37-7.68 (m, 10H, Ar-H), 5.87 (s, 2H, CH_2_); ^13^C-NMR (DMSO-*d*_6_): *δ* 165.90, 164.56, 157.99, 145.50, 140.17, 133.53, 133.23, 131.97, 131.25, 131.18, 120.08, 129.54, 129.09, 128.61, 128.36, 55.41; IR: *ν* 3066.8, 3026.3, 2966.5, 1676.1 cm^-1^; Anal. Calc. for C_19_H_12_Cl_2_N_4_OS_2_: C 51.01%, H 2.70%, N 12.52%. Found: C 51.49%, H 2.93%, N 12.82%.

*5-Chloro-2-[[5-(2-chlorophenyl)-1,3,4-oxadiazol-2-ylthio]methyl]-6-phenylpyridazin-3(2H)-one* (**6e**): Yellow solid, yield 80%, m.p. 112.9-114.7 °C; ^1^H-NMR (CDCl_3_): *δ* 7.33-7.87 (m, 9H, Ph-H), 7.14 (s, 1H, pyridazine-H), 5.83 (s, 2H, CH_2_); ^13^C-NMR (CDCl_3_): *δ* 165.15, 162.32, 158.13, 146.31, 140.92, 133.22, 132.91, 132.64, 131.33, 131.16, 129.81, 129.18, 128.78, 128.28, 127.14, 122.73, 53.27; IR: *ν* 3061.0, 3041.7, 2980.0, 1678.0 cm^-1^; Anal. Calc. for C_19_H_12_Cl_2_N_4_O_2_S: C 52.91%, H 2.80%, N 12.99%. Found: C 52.41%, H 3.29%, N 12.72%. 

*5-Chloro-2-[[5-(2-methoxyphenyl)-1,3,4-thiadiazol-2-ylthio]methyl]-6-phenylpyridazin-3(2H)-one* (**6f**): Yellow solid, yield 75%, m.p. 123.6-125.8 °C; ^1^H-NMR (CDCl_3_): *δ* 7.14-8.49 (m, 10H, Ar-H), 3.93 (s, 3H,OCH_3_), 5.84 (s, 2H, CH_2_); ^13^C-NMR (CDCl_3_): *δ* 164.62, 162.25, 158.19, 155.88, 145.75, 140.62, 133.06, 132.47, 129.64, 129.23,128.78, 128.44, 128.19, 121.42, 118.96, 111.32, 55.76, 55.25; IR (KBr): *ν* 3061.0, 3028.2, 2937.6, 1670.3 cm^-1^; Anal. Calc. for C_20_H_15_ClN_4_O_2_S_2_: C 54.23%, H 3.41%, N 12.65%. Found: C 54.81%, H 3.02%, N 12.92%.

*5-Chloro-2-[[5-(2-methoxyphenyl)-1,3,4-oxadiazol-2-ylthio]methyl]-6-phenylpyridazin-3(2H)-one* (**6g**): Light yellow solid, yield 70%, m.p. 89.2-90.6 °C; ^1^H-NMR (CDCl_3_): *δ* 6.9-7.8 (m, 10H, Ar-H), 3.88 (s, 3H, CH_3_), 5.80 (s, 2H, CH_2_); ^13^C-NMR (CDCl_3_): *δ* 165.63, 161.25, 158.14, 157.90, 146.17, 140.84, 133.39, 132.93, 130.49, 129.74, 129.21, 128.76, 128.25, 120.79, 112.56, 111.95, 56.02, 53.43; IR: *ν* 3070.7, 3034.0, 2983.8,1664.6 cm^-1^; Anal. Calc. for C_20_H_15_ClN_4_O_3_S: C 56.27%, H 3.54%, N 13.12%. Found: C 56.69%, H 3.92%, N 13.60%.

*5-Chloro-2-[[5-(2,4-dichlorophenyl)-1,3,4-thiadiazol-2-ylthio]methyl]-6-phenylpyridazin-3(2H)-one* (**6h**): Light yellow crystals, yield 75%, m.p. 142.5-144.1 °C; ^1^H-NMR (CDCl_3_): *δ* 7.36-8.26 (m, 8H, Ph-H), 7.14 (s, 1H, pyridazine-H), 5.88 (s, 2H, CH_2_); ^13^C-NMR (CDCl_3_): *δ* 164.90, 164.09, 158.20, 146.02, 140.79, 137.53, 133.13, 133.00, 131.65, 130.41, 129.81, 129.20, 128.83, 128.30, 128.04, 127.33, 54.59; IR: *ν* 3068.7, 3022.4, 2953.0, 1674.2 cm^-1^; Anal. Calc. for C_19_H_11_C_l3_N_4_OS_2_: C 47.36%, H 2.30%, N 11.63%. Found: C 47.29%, H 2.68%, N 11.30%.

*5-Chloro-2-[[5-(2,4-dichlorophenyl)-1,3,4-oxadiazol-2-ylthio]methyl]-6-phenylpyridazin-3(2H)-one* (**6i**): White solid, yield 73%, m.p. 134.5-136.1 °C; ^1^H-NMR (DMSO-*d*_6_): *δ* 7.36-7.93 (m, 9H, Ar-H), 5.87 (s, 2H, CH_2_); ^13^C-NMR (DMSO-*d*_6_): *δ* 163.96, 162.63, 158.04, 145.74, 140.22, 137.82, 133.50, 133.35, 132.75, 131.34, 130.06, 129.52, 129.07, 128.59, 121.44, 53.85; IR: *ν* 3057.1, 3026.3, 2933.7, 1676.1 cm^-1^; Anal. Calc. for C_19_H_11_Cl_3_N_4_O_2_S: C 49.00%, H 2.38%, N 12.03%. Found: C 48.73%, H 2.69%, N 12.39%.

### 3.7. Preparation of 5-chloro-6-phenyl-2-[(5-substituted-phenyl)-1,3,4-thiadiazol-2-ylsulfonyl]methyl) pyridazin-3(2H)-ones ***7a-7c***

To a mixture of compound **6** (1.1 mmol) and ethanol (5 mL) was added 30% H_2_O_2_ (5.5 mmol) and (NH_4_)_6_Mo_7_O_24_ (0.011 mmol). The mixture was stirred at 40 °C for 18 h and then filtered off to give a crude product, which was recrystallized from a mixture of anhydrous ethanol and DMF (v/v = 3:1) to afford the desired products. 

*5-Chloro-6-phenyl-2-[[5-(3,4,5-trimethoxyphenyl)-1,3,4-thiadiazol-2-ylsulfonyl]methyl]pyridazin-3(2H)-one* (**7a**): Yellow solid, yield 45%, m.p. 143.3-145.0 °C; ^1^H-NMR (CDCl_3_): *δ* 7.11-7.42 (m, 8H, Ar-H), 5.90 (s, 2H, CH2), 3.94 (s, 9H, CH3); ^13^C-NMR (CDCl_3_): *δ* 174.34, 165.74, 158.15, 153.86, 147.10, 142.17, 141.28, 132.45, 130.01, 129.07, 129.01, 128.30, 123.66, 105.82, 69.11, 61.23, 56.56; IR: *ν* 3066.8, 300.2, 2837.6, 2839.2, 1689.6 cm^-1^; Anal. Calc. for C_22_H_19_ClN_4_O_6_S_2_: C 49.39%, H 3.58%, N 10.47%. Found: C 52.94%, H 3.29%, N 11.62%.

*5-chloro-6-phenyl-2-[(5-phenyl-1,3,4-oxadiazol-2-ylsulfonyl)methyl]pyridazin-3(2H)-one* (**7b**): White solid, yield 55%, m.p. 166-168 °C; ^1^H-NMR (DMSO-*d*_6_): *δ* 7.35-8.00 (m, 11H, Ar-H), 6.31 (s, 2H, CH_2_); ^1^H-NMR (DMSO-*d*_6_): *δ* 167.15, 160.93, 158.48, 146.91, 140.80, 133.02, 130.33, 130.14, 129.48, 129.32, 128.65, 128.01, 122.35, 69.39; IR: *ν* 3095.7, 2999.3, 2914.4, 1672.3 cm^-1^; Anal. Calc. for C_19_H_13_ClN_4_O_4_S: C 57.50%, H 3.30%, N 14.14%. Found: C 7.41%, H 3.54%, N 14.64%.

*5-chloro-6-phenyl-2-[(5-phenyl-1,3,4-thiadiazol-2-ylsulfonyl)methyl]pyridazin-3(2H)-one* (**7c**): White solid, yield 60%, m.p. 154-156 °C; ^1^H-NMR (DMSO-*d*_6_): *δ* 7.37-8.03 (m, 11H, Ar-H), 6.20 (s, 2H, CH_2_); ^13^C-NMR (DMSO-*d*_6_): *δ* 175.00, 166.00, 159.00, 158.26, 147.00, 140.50, 133.48, 133.05, 130.23, 130.12, 129.39, 128.93, 128.60, 69.36; IR: *ν* 3089.6, 3001.2, 2902.8, 1678.1 cm^-1^; Anal. Calc. for C_19_H_13_ClN_4_O_3_S_2_: C 51.29%, H 2.95%, N 12.59%. Found: C 51.63%, H 2.69%, N 12.92%.

### 3.8. Antifungal bioassays

The antifungal activity of all synthesized compounds was tested against *F. oxysporum, G. zeae,* and *C. mandshurica* by the poison plate technique [31]. All the compounds were dissolved in DMSO (10 mL) before mixing with Potato Dextrose Agar (PDA, 90 mL). The final concentration of the compounds in the medium was fixed at 50 μg/mL. The three kinds of fungi were incubated in PDA at 25 ± 1 °C for 5 days to get new mycelium for the antifungal assays, and then a mycelia disk of approximately 0.45 cm diameter cut from the culture medium was picked up with a sterilized inoculation needle and inoculated in the center of PDA plate. The inoculated plates were incubated at 25 ± 1 °C for 5 days. DMSO in sterilized distilled water served as control, while hymexazole was used as positive control for each treatment with three replicates being conducted for each experiment. The radial growth of the fungal colonies was measured on the sixth day and the data were statistically analyzed. The in vitro inhibiting effects of the test compounds on the fungi were calculated by the formula CV = (A − B)/A, where A represents the diameter of fungi growth on untreated PDA, B represents the diameter of fungi on treated PDA, and CV represents the rate of inhibition.

## 4. Conclusions

In the present study, a mild and effective method for the preparation of 21 novel pyridazine derivatives were undertaken by employing mucochloric acid and benzene as the starting materials. The synthesized compounds were characterized by spectral data (^1^H-NMR, ^13^C-NMR, IR) and elemental analysis. The compounds were subjected to fungicidal activities *in vitro* against *G. zeae*, *F. oxysporum* and *C. mandshurica*. The results showed that the synthesized pyridazine compounds possessed weak to good antifungal activities against the tested fungi, among which, compounds **3e**, **3h**, **7b**, **7c** displayed good antifungal activities. Further studies are currently underway to establish a definite structure activity relationship. 
